# Supplementary material for: Mechanisms of urate transport and uricosuric drugs inhibition in human URAT1
Source: Nat Commun. 2025 Feb 10;16:1512. doi: 10.1038/s41467-025-56843-5 (PMC11811179; doi:10.1038/s41467-025-56843-5)
Supplement: Supplementary file 1 — Supplementary Information [file 41467_2025_56843_MOESM1_ESM.pdf]

Supplementary Information

Mechanisms of urate transport and uricosuric drugs inhibition in human URAT1

Wenjun Guo[1,2,3,4,#], Miao Wei [1,#], Yunfeng Li [5], Jiaxuan Xu[1], Jiahe Zang [1],

Yuezhou Chen [2,3,5], and Lei Chen [1,2,3,4,*]

[1] State Key Laboratory of Membrane Biology, College of Future Technology, Institute of Molecular Medicine, Peking. University, Beijing Key Laboratory of Cardiometabolic Molecular Medicine, Beijing 100871, China.

[2] Academy for Advanced Interdisciplinary Studies, Peking University, Beijing 100871, China

[3] Peking-Tsinghua Center for Life Sciences, Peking University, Beijing 100871, China

[4] National Biomedical Imaging Center, Peking University, Beijing, 100871, China

[5] MOE Key Laboratory of Cell Proliferation and Differentiation, School of Life Sciences, Peking University, Beijing 100871, China.

[#] These authors contribute equally to this work

[*]Correspondence: Lei Chen, chenlei2016@pku.edu.cn

Supplementary Table 1

Supplementary Figures 1-10

Supplementary Table 1

Cryo-EM data collection, refinement and validation statistics

PDB ID<br>EMDB ID	Urate-bound hURAT1$_{EM}$<br>9IRW<br>EMD-60823	Benzbromarone-bound hURAT1$_{EM}$<br>9IRX<br>EMD-60824	Verinurad-bound hURAT1$_{EM}$<br>9IRY<br>EMD-60825
**Data collection and processing**			
Magnification	81,000 ×	81,000 ×	81,000 ×
Voltage (kV)	300	300	300
Electron exposure (e^{-}/Å^{2})	70	70	70
Defocus range (μm)	-1.5 to -1.8	-1.5 to -1.8	-1.5 to -1.8
Pixel size (Å)	1.067	1.067	1.067
Symmetry imposed	*C1*	*C1*	*C1*
Initial particle images (no.)	6,243,714	7,177,004	5,374,146
Final particle images (no.)	310,852	680,885	659,248
Map resolution (Å)	3.3	3.0	3.2
FSC threshold	0.143	0.143	0.143
Map resolution range (Å)	250-3.3	250-3.0	250-3.2
**Refinement**			
Initial model used (PDB code)			
Model resolution (Å)	3.4	3.1	3.4
FSC threshold	0.5	0.5	0.5
Model resolution range (Å)	250-3.4	250-3.1	250-3.4
Map sharpening *B* factor (Å^{2})	-163.0	-161.3	-164.2
Model composition			
Non-hydrogen atoms	3,808	3,509	3,146
Protein residues	499	469	421
Ligands	1	1	1
B factors (Å^{2})			
Protein	51.77	36.26	33.07
Ligand	49.60	38.13	51.91
R.m.s. deviations			
Bond lengths (Å)	0.006	0.004	0.006
Bond angles (°)	1.070	0.962	1.011
Validation			
MolProbity score	2.22	1.45	1.66
Clashscore	8.80	6.63	10.58
Poor rotamers (%)	3.47	0.56	1.25
Ramachandran plot			
Favored (%)	95.09	97.59	97.81
Allowed (%)	4.91	2.41	2.19
Disallowed (%)	0.00	0.00	0.00

Supplementary Fig.1

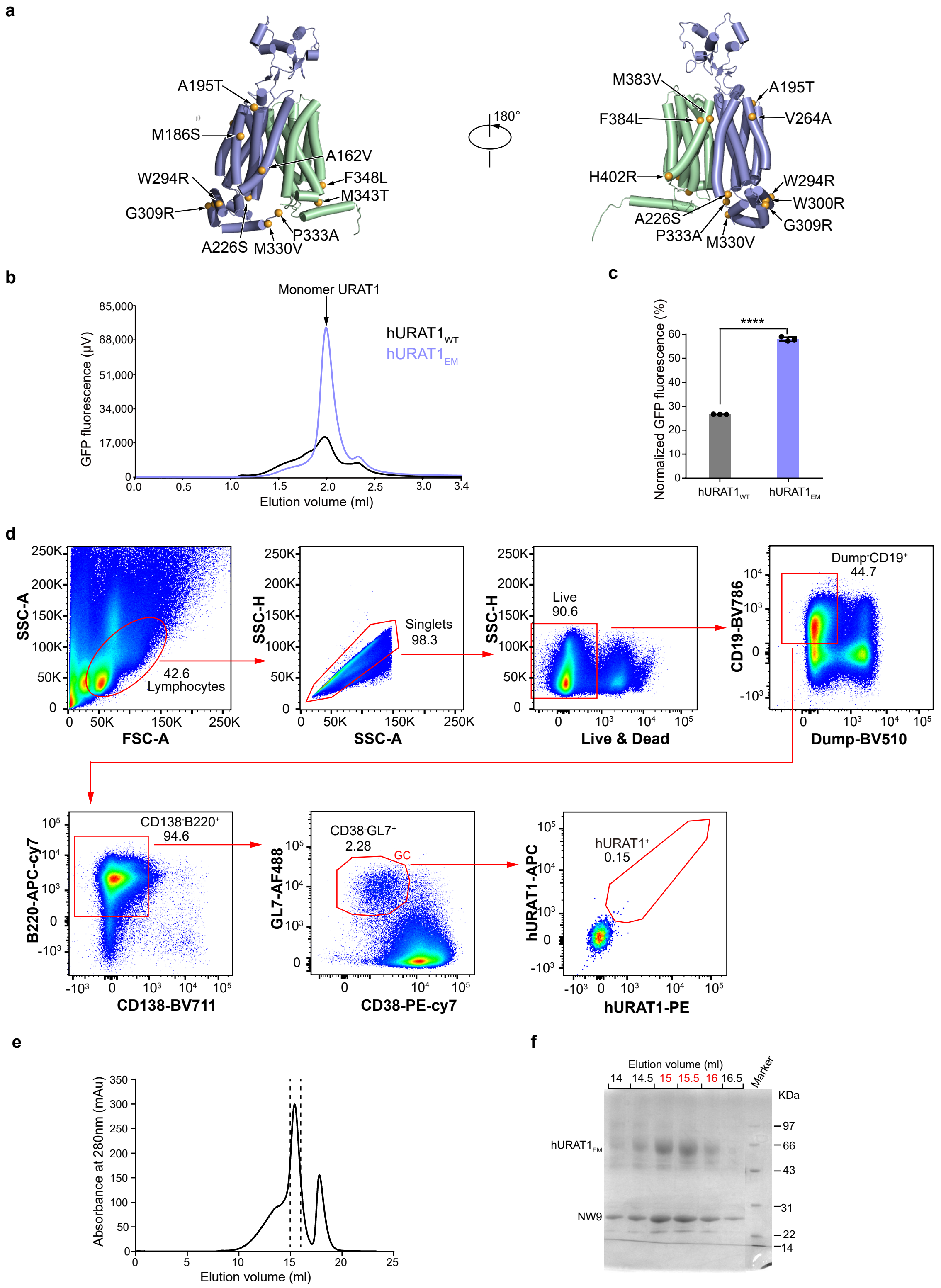

Supplementary Fig. 1. Consensus design and protein purification of hURAT1. a, Distribution of the consensus mutations of hURAT1$_{EM}$, mapped on the predicted structure of hURAT1. **b,** Representative FSEC profile of hURAT1$_{WT}$ and hURAT1$_{EM}$ cell lysates solubilized by 1%MNG with 0.1% CHS. **c,** Thermostability of hURAT1$_{WT}$ and hURAT1$_{EM}$ cell lysates. The GFP fluorescence of hURAT1 monomer peak after heating is normalized to that without heating. Data are shown as means ± s.d.; $n = 3$ technical replicates. The experiment was performed independently twice with similar results. Two-tailed Student's t test. *****P* < 0.0001. **d,** Representative FACS plot for sorting strategies to detect hURAT1$_{EM}$-binding cells in splenic germinal center. Single live PE and APC double positive germinal center B cells ($CD4^-CD8a^-CD19^+CD138^-B220^+CD38^-GL7^+$) were sorted as single cell into 96-well plates for single B cell RT-PCR. The dump channel includes CD4 and CD8a. **e,** Size-exclusion chromatography profile of the hURAT1$_{EM}$ in nanodiscs. Fractions between dashed lines were used for cryo-EM sample preparation. **f,** Coomassie brilliant blue-stained SDS-PAGE gel of the purified hURAT1$_{EM}$ in nanodiscs. The experiments were repeated independently three times with similar results. The fractions colored in red were used for cryo-EM sample preparation.

Supplementary Fig.2

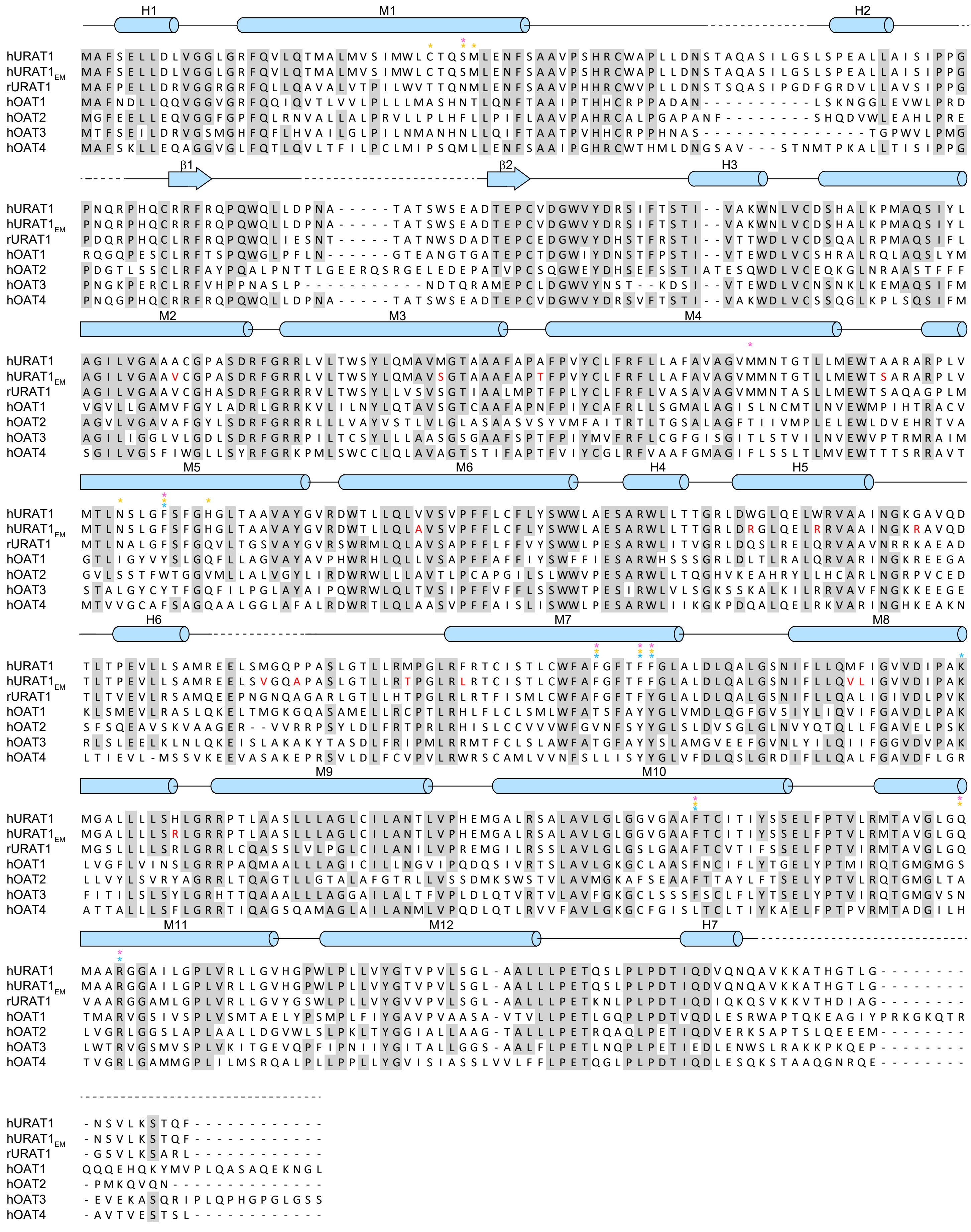

Supplementary Fig. 2. Sequence alignment among hURAT1, hURAT1EM and other related SLC22 family proteins. The sequence alignment of human URAT1 (hURAT1), the cryo-EM construct (hURAT1$_{EM}$), rat URAT1 (rURAT1), human OAT1 (hOAT1), human OAT2 (hOAT2), human OAT3 (hOAT3), and human OAT4 (hOAT4). Conserved residues are shaded in grey. Secondary structures are shown above and colored in blue. Residues interacting with urate, benzbromarone and verinurad are denoted by * and colored in cyan, yellow and pink, respectively. Mutations of hURAT1$_{EM}$ are colored in red.

Supplementary Fig.3

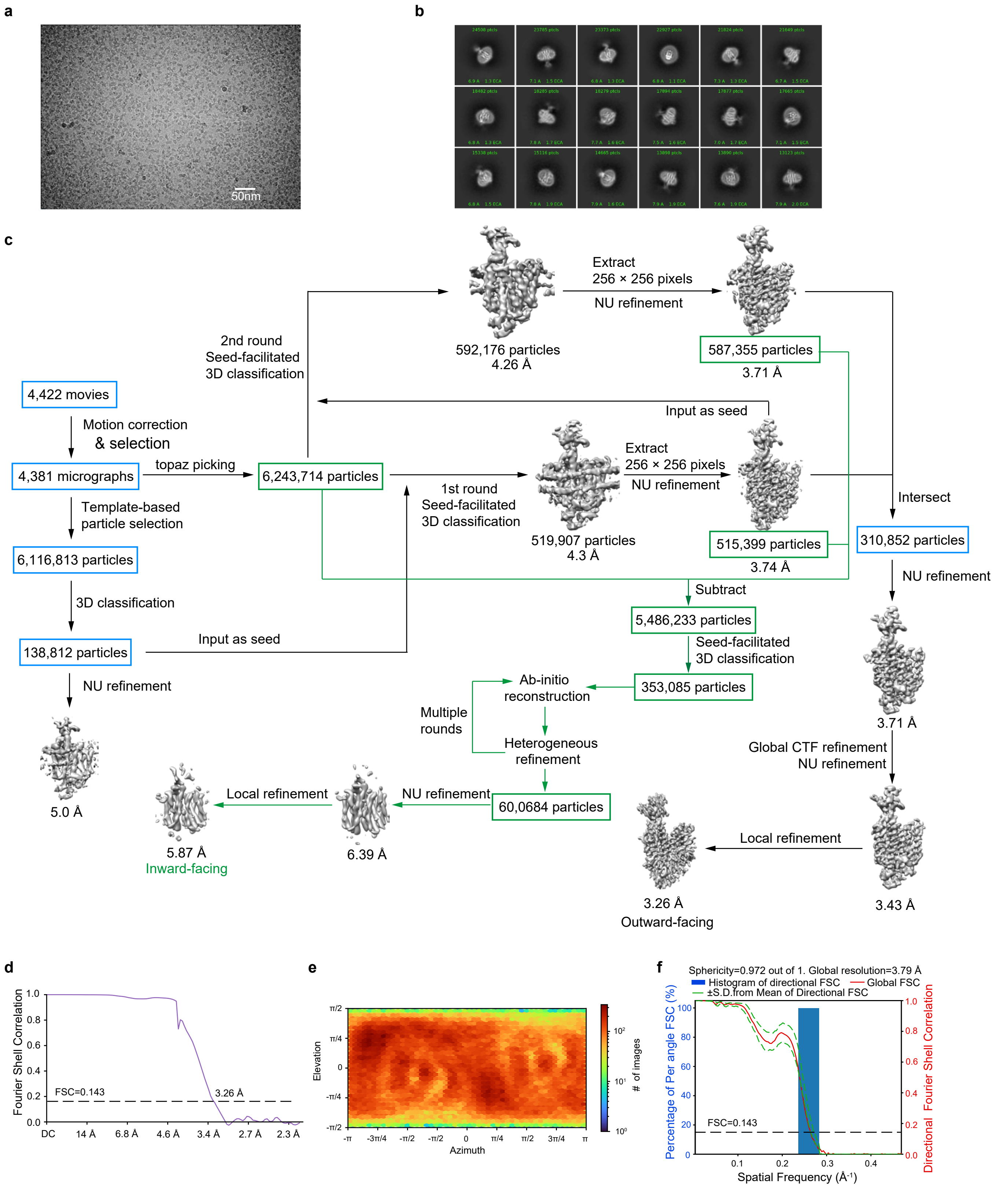

Supplementary Fig. 3. Cryo-EM image analysis of urate-bound hURAT1. a, Representative raw micrograph of urate-bound hURAT1 in nanodiscs. Scale bar, 50 nm. **b,** 2D class averages of urate-bound hURAT1. **c,** Workflow of the cryo-EM data processing. **d,** Gold-standard Fourier Shell Correlation (FSC) of the cryo-EM map. Estimation of resolution was based on the criterion of FSC 0.143 cut-off. **e,** Angular distribution of all particles contributed to the final 3D map. **f,** Histogram of directional FSC curves. Individual 1D FSC curves are compiled into 3D FSC and represented within a histogram. The spread of the directional resolutions defines by plus and minus one standard deviation from the mean of the directional resolutions.

Supplementary Fig.4

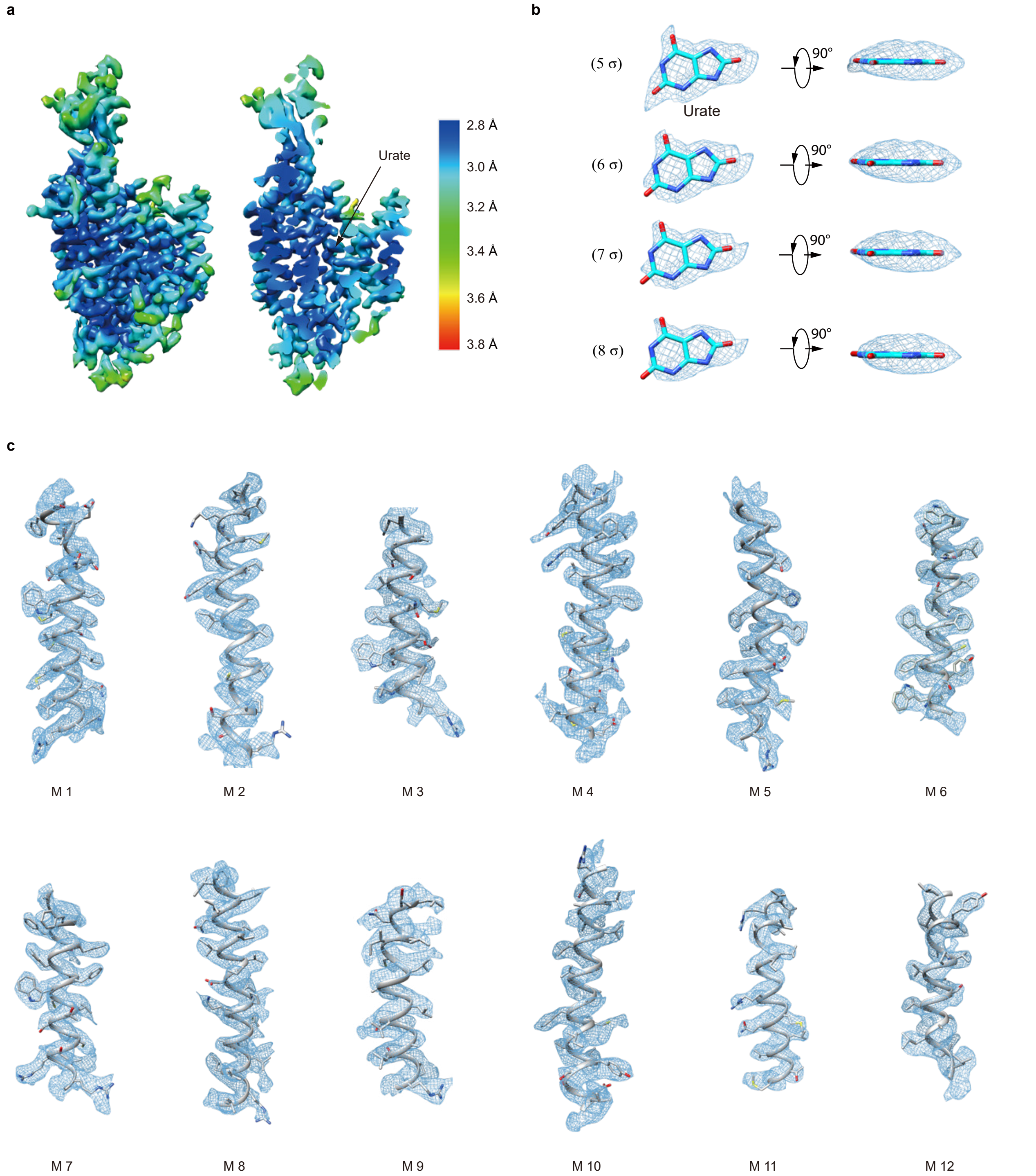

Supplementary Fig. 4. Cryo-EM densities of urate-bound hURAT1. a, Side view and a cut-open view of the local resolution distribution of the hURAT1 in complex with urate. **b,c,** Electron density maps (blue mesh) of urate **(b)** (contoured at 5 σ, 6 σ,7 σ and 8σ, shown in the parenthesis) and transmembrane helices **(c)** (contoured at 8 σ) superimposed with the atomic model.

Supplementary Fig.5

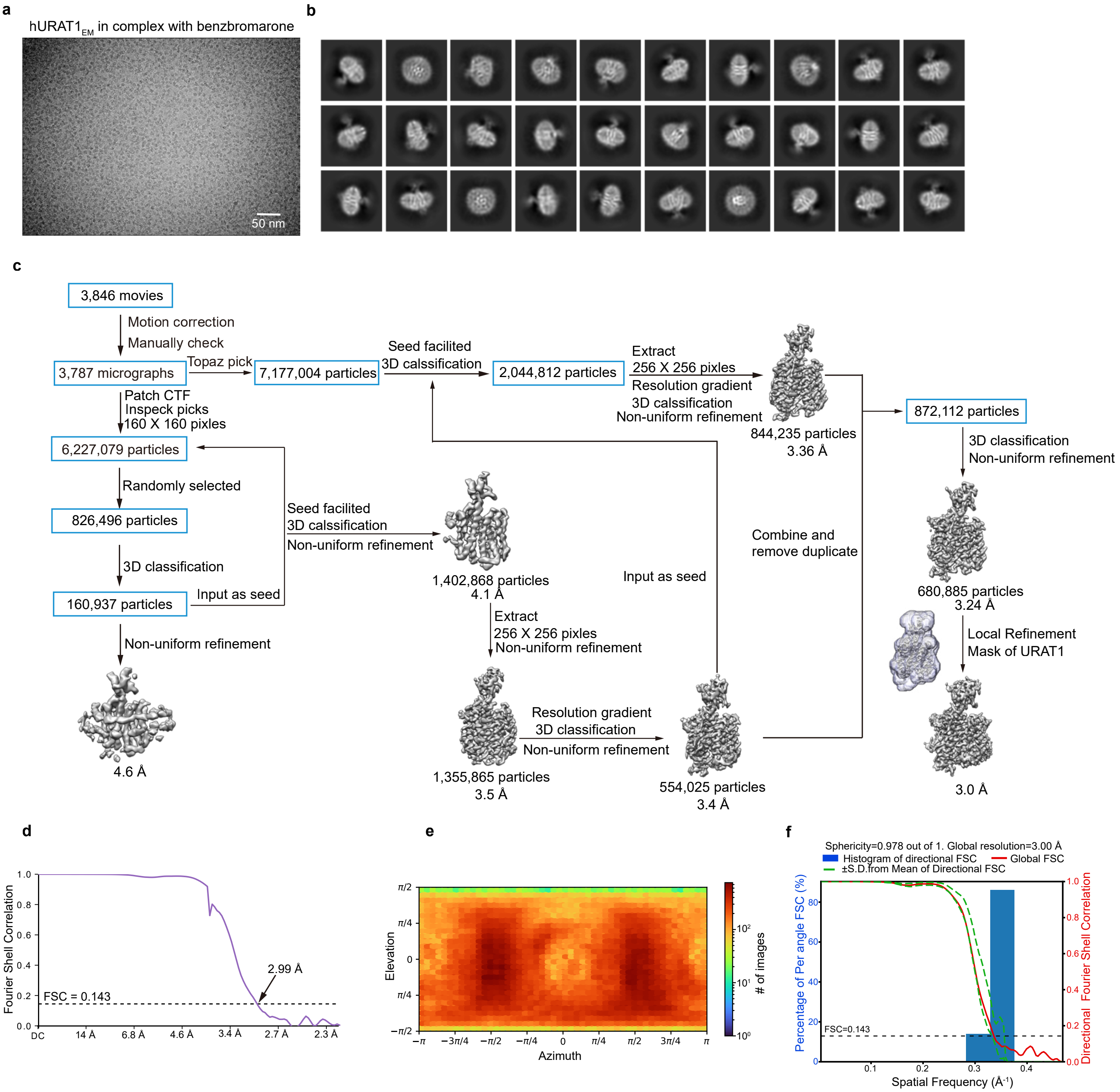

Supplementary Fig. 5. Cryo-EM image processing of hURAT1 in complex with benzbromarone. a, Representative raw micrograph of benzbromarone-bound hURAT1 in nanodiscs. Scale bar, 50 nm. **b,** 2D class averages of benzbromarone-bound hURAT1 sample. **c,** Workflow of the cryo-EM data processing. **d,** Gold-standard FSC of the cryo-EM map. Estimation of resolution was based on the criterion of FSC 0.143 cut-off. **e,** Angular distribution of all particles contributed to the final 3D map. **f,** Histogram of directional FSC curves. Individual 1D FSC curves are compiled into 3D FSC and represented within a histogram. The spread of the directional resolutions defines by plus and minus one standard deviation from the mean of the directional resolutions.

Supplementary Fig.6

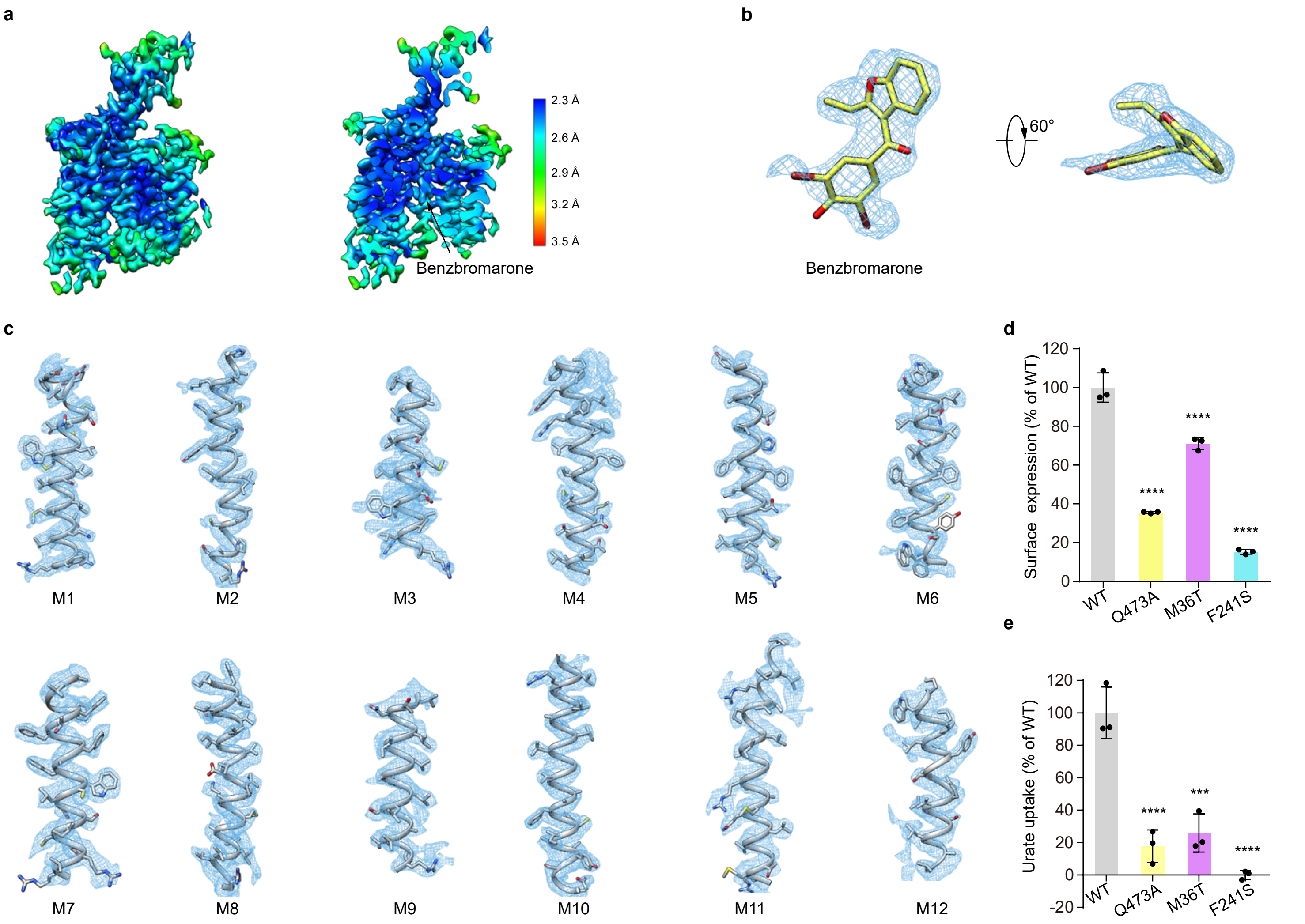

Supplementary Fig. 6. Representative electron density maps of benzbromarone-bound hURAT1. a, Side view and a cut-open view of the local resolution distribution of the hURAT1 in complex with benzbromarone. **b, c,** Electron density maps of benzbromarone and the transmembrane helices (M1-M12) are shown as blue meshes, and contoured at 7 σ. **d,** Surface expression of various mutants of hURAT1. Data are normalized to hURAT1$_{WT}$, shown as means ± s.d.; *n* = 3 technical replicates. The experiment was performed independently twice with similar results. Statistical significance compared with hURAT1$_{WT}$ was determined using ordinary one-way ANOVA with Dunnett's multiple comparisons test. ****$P < 0.0001$. **e,** Urate uptake activities of various mutants of hURAT1. Data are normalized to hURAT1$_{WT}$, shown as means ± s.d.; *n* = 3 technical replicates. The experiment was performed independently twice with similar results. Statistical significance compared with hURAT1$_{WT}$ was determined using ordinary one-way ANOVA with Dunnett's multiple comparisons test, ***$P < 0.001$; ****$P < 0.0001$. *P* value between hURAT1$_{WT}$ and mutant M36T is 0.0001.

Supplementary Fig.7

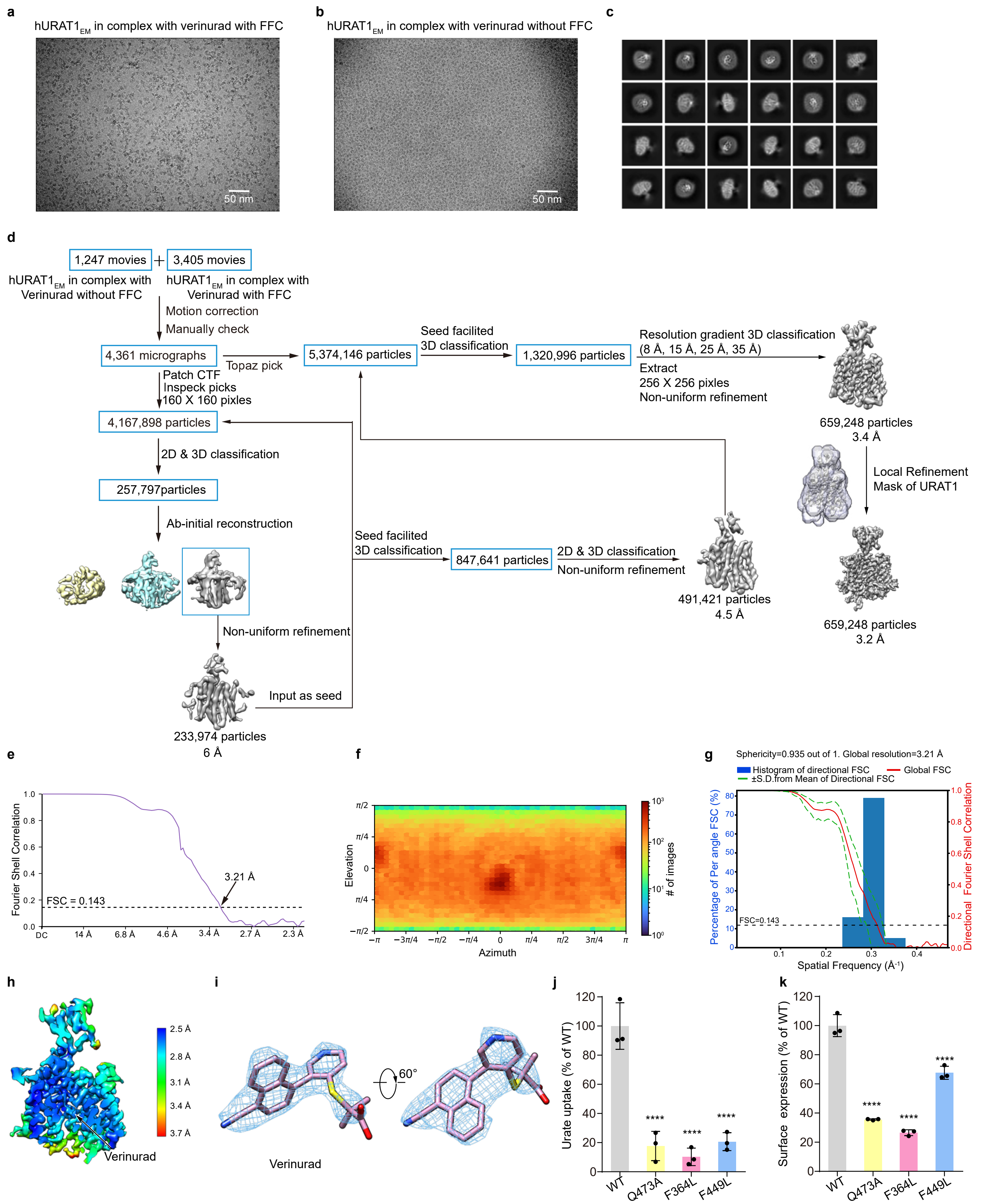

Supplementary Fig. 7. Cryo-EM image processing of hURAT1 in complex with verinurad. a,b, Representative raw micrograph of verinurad-bound hURAT1 in nanodiscs with FFC or without FFC. Scale bar, 50 nm. **c,** 2D class averages of verinurad-bound hURAT1 sample. **d,** Workflow of the cryo-EM data processing. **e,** Gold-standard FSC of the cryo-EM map. Estimation of resolution was based on the criterion of FSC 0.143 cut-off. **f,** Angular distribution of all particles contributed to the final 3D map. **g,** Histogram of directional FSC curves. Individual 1D FSC curves are compiled into 3D FSC and represented within a histogram. The spread of the directional resolutions defines by plus and minus one standard deviation from the mean of the directional resolutions. **h,** A cut-open view of the local resolution distribution of the hURAT1 in complex with verinurad. **i,** Electron density map of verinurad is shown in blue meshes, and contoured at 14 σ. **j,** Urate uptake activities of various mutants of hURAT1. Data are normalized to $hURAT1_{WT}$, shown as means ± s.d.; *n* = 3 technical replicates. The experiment was performed independently twice with similar results. Statistical significance compared with $hURAT1_{WT}$ was determined using ordinary one-way ANOVA with Dunnett's multiple comparisons test, *****P* < 0.0001. Data for $hURAT1_{WT}$ and Q473A are the same as shown in **Supplementary Fig. 6e**. **k,** Surface expression of various mutants of hURAT1. Data are normalized to $hURAT1_{WT}$, shown as means ± s.d.; *n* = 3 technical replicates. The experiment was performed independently twice with similar results. Statistical significance compared with $hURAT1_{WT}$ was determined using ordinary one-way ANOVA with Dunnett's multiple comparisons test. *****P* < 0.0001. Data for $hURAT1_{WT}$ and Q473A are the same as shown in **Supplementary Fig. 6d**.

Supplementary Fig.8

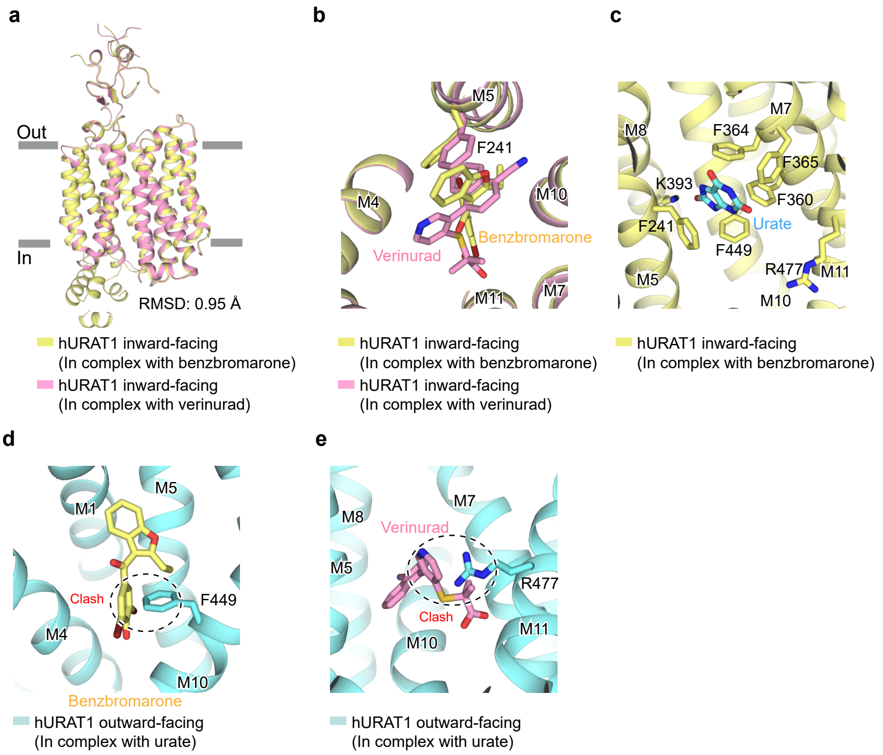

Supplementary Fig. 8. Structure comparations among benzbromarone-bound, verinurad-bound and urate-bound hURAT1. a, Superposition of benzbromarone-bound and verinurad-bound hURAT1. Benzbromarone-bound hURAT1 is colored in yellow and verinurad-bound hURAT1 is colored in pink. **b,** The structure of verinurad-bound hURAT1 (pink) is overlaid onto the benzbromarone-bound hURAT1 (yellow). Ligands and F241 are shown as sticks. **c,** The ligand urate (cyan) is overlaid onto the benzbromarone-bound hURAT1 (yellow) in inward-facing conformation. Urate and related residues are shown as sticks. **d, e,** The ligands benzbromarone (yellow) and verinurad (pink) are overlaid onto the urate-bound hURAT1 (cyan) in outward-facing conformation. Ligands and related residues are shown as sticks. Steric clashes between ligands and residues are denoted with dashed circles.

Supplementary Fig.9

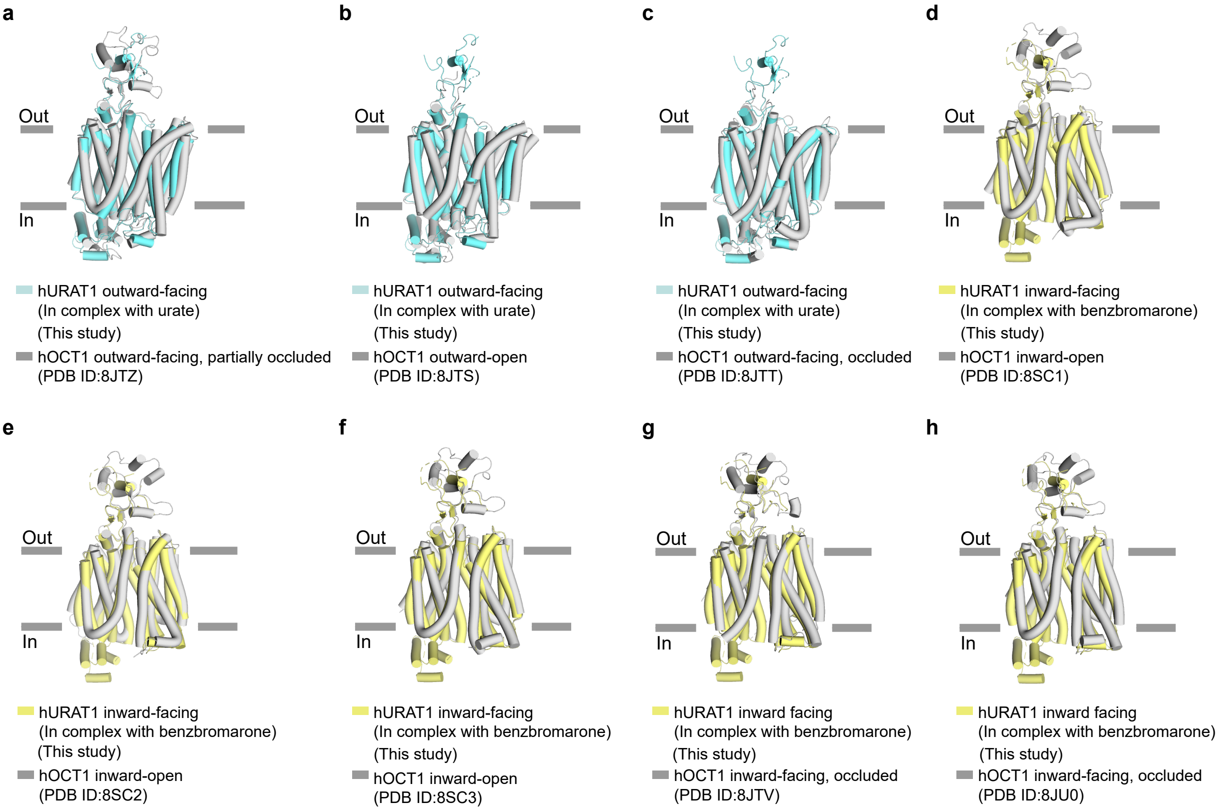

Supplementary Fig. 9. Structure comparations among hURAT1s and hOCT1s. a-c, Structural superposition of hURAT1 (cyan) and hOCT1s (grey) in outward-facing conformation. **d-h**, Structural superposition of hURAT1 (yellow) and hOCT1s (grey) in inward-facing conformation.

Supplementary Fig. 10

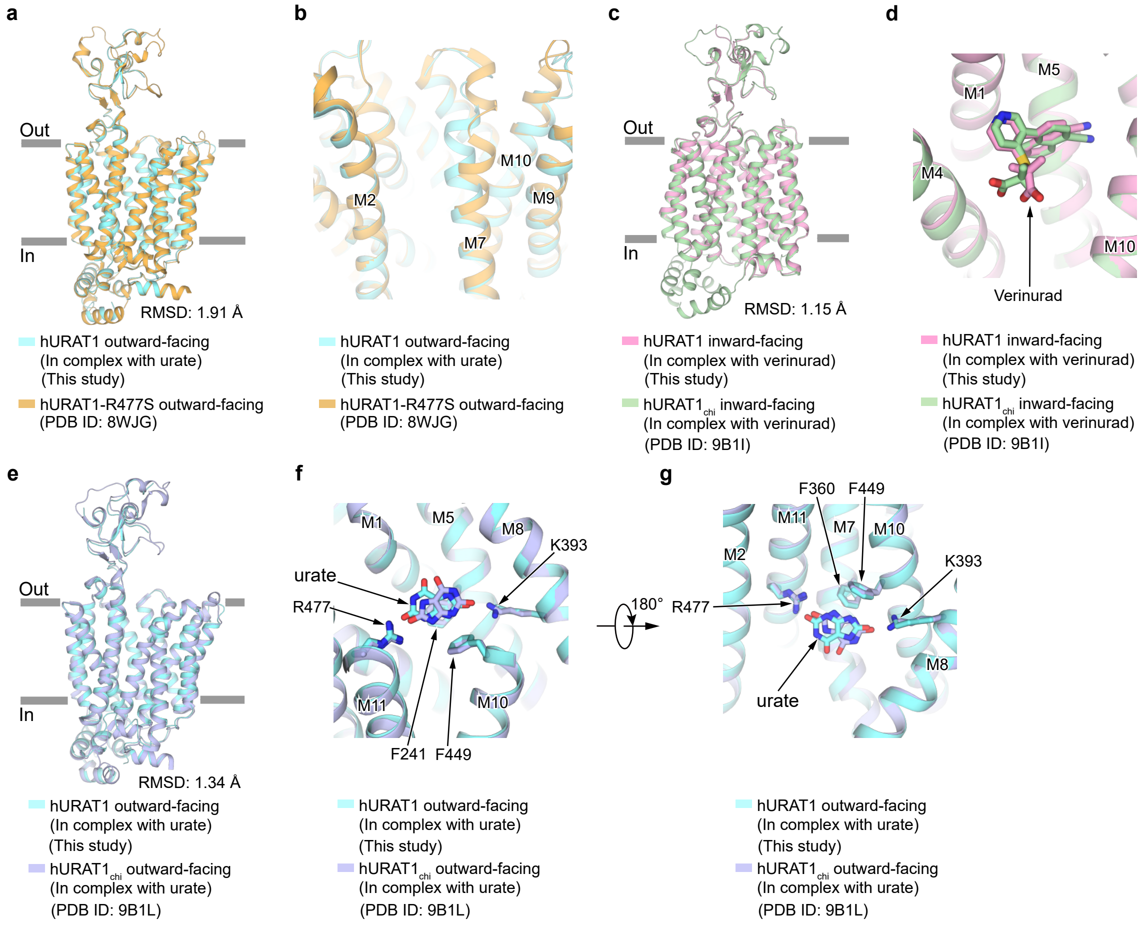

Supplementary Fig. 10. Structure comparations among hURAT1s. a, Superposition of urate-bound hURAT1 (cyan) and hURAT1-R477S (orange). **b,** The structure of urate-bound hURAT1 (cyan) is overlaid onto the hURAT1-R477S (orange). **c,** Superposition of verinurad-bound hURAT1 (pink) and verinurad-bound hURAT1$_{chi}$ (green). **d,** The structure of verinurad-bound hURAT1 (pink) is overlaid onto the verinurad-bound hURAT1$_{chi}$ (green). Ligands are shown as sticks. **e,** Superposition of urate-bound hURAT1 (cyan) and urate-bound hURAT1$_{chi}$ (purple). **f, g,** The structure of urate-bound hURAT1 (cyan) is overlaid onto the urate-bound hURAT1$_{chi}$ (purple). Urate and its interacting residues are shown as sticks.
