# Supplementary material for: Mechanisms of urate transport and uricosuric drugs inhibition in human URAT1
Source: Nat Commun. 2025 Feb 10;16:1512. doi: 10.1038/s41467-025-56843-5 (PMC11811179; doi:10.1038/s41467-025-56843-5)
Supplement: Supplementary file 2 — Description of Additional Supplementary Files [file 41467_2025_56843_MOESM2_ESM.pdf]

Description of Additional Supplementary Files

File Name: Supplementary Movie 1
Description: **Structural changes of hURAT1 from the outward-facing conformation to the inward-facing conformation.**
hURAT1 structure is morphed between the outward-facing (urate-bound) state and the inward-facing (benzbromaronebound) state. Key residues are shown in sticks.
