# Supplementary material for: Mechanisms of urate transport and uricosuric drugs inhibition in human URAT1
Source: Nat Commun. 2025 Feb 10;16:1512. doi: 10.1038/s41467-025-56843-5 (PMC11811179; doi:10.1038/s41467-025-56843-5)
Supplement: Supplementary file 4 — Reporting Summary [file 41467_2025_56843_MOESM4_ESM.pdf]

nature portfolio

Corresponding author(s): Lei Chen

Last updated by author(s): 2024-12-30

Reporting Summary

Nature Portfolio wishes to improve the reproducibility of the work that we publish. This form provides structure for consistency and transparency in reporting. For further information on Nature Portfolio policies, see our Editorial Policies and the Editorial Policy Checklist.

Statistics

For all statistical analyses, confirm that the following items are present in the figure legend, table legend, main text, or Methods section.

n/a	Confirmed	
☐	☒	The exact sample size (*n*) for each experimental group/condition, given as a discrete number and unit of measurement
☐	☒	A statement on whether measurements were taken from distinct samples or whether the same sample was measured repeatedly
☐	☒	The statistical test(s) used AND whether they are one- or two-sided<br>*Only common tests should be described solely by name; describe more complex techniques in the Methods section.*
☒	☐	A description of all covariates tested
☒	☐	A description of any assumptions or corrections, such as tests of normality and adjustment for multiple comparisons
☐	☒	A full description of the statistical parameters including central tendency (e.g. means) or other basic estimates (e.g. regression coefficient) AND variation (e.g. standard deviation) or associated estimates of uncertainty (e.g. confidence intervals)
☐	☒	For null hypothesis testing, the test statistic (e.g. *F*, *t*, *r*) with confidence intervals, effect sizes, degrees of freedom and *P* value noted<br>*Give P values as exact values whenever suitable.*
☒	☐	For Bayesian analysis, information on the choice of priors and Markov chain Monte Carlo settings
☒	☐	For hierarchical and complex designs, identification of the appropriate level for tests and full reporting of outcomes
☒	☐	Estimates of effect sizes (e.g. Cohen's *d*, Pearson's *r*), indicating how they were calculated

Our web collection on statistics for biologists contains articles on many of the points above.

Software and code

Policy information about availability of computer code

Data collection	EPU-2.12.1.2782REL
Data analysis	MotionCor2-1.3.2, cryoSPARC-4.5.3, PHENIX1.19.2, Coot-0.9.8.93, UCSF Chimera-1.15, ChimeraX-1.2.5, Graphpad Prism v6.0, Pymol-1.7.0.5, Pymol-2.6.0, BioEdit-7.2.5, Microsoft Excel-2013, ChemDraw 18.0

For manuscripts utilizing custom algorithms or software that are central to the research but not yet described in published literature, software must be made available to editors and reviewers. We strongly encourage code deposition in a community repository (e.g. GitHub). See the Nature Portfolio guidelines for submitting code & software for further information.

Data

Policy information about availability of data

All manuscripts must include a data availability statement. This statement should provide the following information, where applicable:

- Accession codes, unique identifiers, or web links for publicly available datasets
- A description of any restrictions on data availability
- For clinical datasets or third party data, please ensure that the statement adheres to our policy

Cryo-EM maps of the hURAT1 in the urate-bound state, the benzbromarone-bound state and verinurad-bound state have been deposited in the Electron Microscopy Data Bank (EMDB) under the ID codes: EMD-60823 [https://www.ebi.ac.uk/pdbe/entry/emdb/EMD-60823] (map of urate-bound state hURAT1); EMD-60824 [https://www.ebi.ac.uk/pdbe/entry/emdb/EMD-60824] (map of benzbromarone-bound state hURAT1); EMD-60825 [https://www.ebi.ac.uk/emdb/EMD-60825] (map of verinurad-bound state hURAT1), respectively. Atomic models of hURAT1 in the urate-bound state, the benzbromarone-bound state and [illegible]

verinurad-bound state have been deposited in the Protein Data Bank (PDB) under the ID codes: 9IRW [https://doi.org/10.2210/pdb9IRW/pdb] (atomic model of urate-bound state hURAT1); 9IRX [https://doi.org/10.2210/pdb9IRX/pdb] (atomic model of benzbromarone-bound state hURAT1), 9IRY [https://doi.org/10.2210/pdb9IRY/pdb] (atomic model of verinurad-bound state hURAT1), respectively. The entry 8SDU [https://doi.org/10.2210/pdb8SDU/pdb] used in this study were downloaded from the PDB.

Research involving human participants, their data, or biological material

Policy information about studies with human participants or human data. See also policy information about sex, gender (identity/presentation), and sexual orientation and race, ethnicity and racism.

Reporting on sex and gender	n/a.
Reporting on race, ethnicity, or other socially relevant groupings	n/a.
Population characteristics	n/a.
Recruitment	n/a.
Ethics oversight	n/a.

Note that full information on the approval of the study protocol must also be provided in the manuscript.

Field-specific reporting

Please select the one below that is the best fit for your research. If you are not sure, read the appropriate sections before making your selection.

[x] Life sciences [ ] Behavioural & social sciences [ ] Ecological, evolutionary & environmental sciences

For a reference copy of the document with all sections, see nature.com/documents/nr-reporting-summary-flat.pdf

Life sciences study design

All studies must disclose on these points even when the disclosure is negative.

Sample size	Sufficient cryo-EM data were collected to achieve adequate map resolutions for model building. All of functional experiments were performed at least twice with three biological samples in order to allow for the calculation of mean values and the standard error of the mean.
Data exclusions	Cryo-EM micrographs with ice or ethane contamination, empty carbon, and poor CTF fit (> 4 Å) were excluded manually. Particles belonging to bad classes were discarded and the data processing flowchart were summarized in Extended Data Figures. These criteria were pre-established and the procedure is a common practise in cryo-EM image analysis.
Replication	All attempts at replication were successful according to the detailed protocol described in the methods section. The numbers of replication were described in figure legends.
Randomization	For cryo-EM 3D refinement, all particles were randomly split into two groups. No group allocation was needed for functional experiments in this study.
Blinding	The investigators were blinded to group allocation during cryo-EM half map generation. Blinding is not relevant for protein structure determination and functional assays because these results are not subjective. Our procedure complies with the common practice in the field.

Reporting for specific materials, systems and methods

We require information from authors about some types of materials, experimental systems and methods used in many studies. Here, indicate whether each material, system or method listed is relevant to your study. If you are not sure if a list item applies to your research, read the appropriate section before selecting a response.

Materials & experimental systems

n/a	Involved in the study	
[ ]	[x]	Antibodies
[ ]	[x]	Eukaryotic cell lines
[x]	[ ]	Palaeontology and archaeology
[ ]	[x]	Animals and other organisms
[x]	[ ]	Clinical data
[x]	[ ]	Dual use research of concern
[x]	[ ]	Plants

Methods

n/a	Involved in the study	
[x]	[ ]	ChIP-seq
[ ]	[x]	Flow cytometry
[x]	[ ]	MRI-based neuroimaging

Antibodies

Antibodies used	The antibodies used in flow cytometry include: from Biolegend, IgG1 (406632, clone: RMG1-1, lot: B422285), CD138 (142519, clone: 281-2, lot: B378402), CD19 (115543, clone: 6D5, lot: B386950), Streptavidin (405207, lot: B380870); from Life Technologies, GL7 (53-5902-82, clone: GL-7 (GL7), lot: 2442288), CD38 (25-0381-82, clone: 90, lot: 2460145), B220 (47-0452-82, clone: RA3-6B2, lot: 2703742), CD4 (69-0041-82, clone: GK1.5, lot: 2629005), CD8a (69-0081-82, clone: 53-6.7, lot: 2446938), IgD (63-5993-82, clone: 11-26c (11-26), lot: 2634835), Streptavidin (12-4317-87, lot: 2514129), all dilution 1: 200 for staining.<br>The antibodies used in surface labeling assay include: Rabbit anti-HA mAb (3724; Cell Signaling Technology); HRP labeled Goat anti-rabbit IgG secondary antibody (31460; Invitrogen)
Validation	All of the antibodies were validated by manufacturers.<br>Alexa Fluor® 700 anti-mouse IgG1 Antibody (https://www.biolegend.com/fr-ch/products/alexa-fluor-700-anti-mouse-igg1-antibody-16586?GroupID=BLG3729).<br>Brilliant Violet 711™ anti-mouse CD138 (Syndecan-1) Antibody (https://www.biolegend.com/fr-ch/products/brilliant-violet-711-anti-mouse-cd138-syndecan-1-antibody-8909).<br>Brilliant Violet 785™ anti-mouse CD19 Antibody (https://www.biolegend.com/fr-ch/products/brilliant-violet-785-anti-mouse-cd19-antibody-7962).<br>APC Streptavidin (https://www.biolegend.com/en-gb/products/apc-streptavidin-1470?GroupID=GROUP23).<br>GL7 Monoclonal Antibody (GL-7 (GL7)), Alexa Fluor™ 488, eBioscience™ (https://www.thermofisher.cn/cn/zh/antibody/product/GL7-Antibody-clone-GL-7-GL7-Monoclonal/53-5902-82).<br>CD38 Monoclonal Antibody (90), PE-Cyanine7, eBioscience™ (https://www.thermofisher.cn/cn/zh/antibody/product/CD38-Antibody-clone-90-Monoclonal/25-0381-82).<br>CD45R (B220) Monoclonal Antibody (RA3-6B2), APC-eFluor™ 780, eBioscience™ (https://www.thermofisher.cn/cn/zh/antibody/product/CD45R-B220-Antibody-clone-RA3-6B2-Monoclonal/47-0452-82).<br>CD4 Monoclonal Antibody (GK1.5), eFluor™ 506, eBioscience™ (https://www.thermofisher.cn/cn/zh/antibody/product/CD4-Antibody-clone-GK1-5-Monoclonal/69-0041-82).<br>CD8a Monoclonal Antibody (53-6.7), eFluor™ 506, eBioscience™ (https://www.thermofisher.cn/cn/zh/antibody/product/CD8a-Antibody-clone-53-6-7-Monoclonal/69-0081-82).<br>IgD Monoclonal Antibody (11-26c (11-26)), Super Bright™ 600, eBioscience™ (https://www.thermofisher.cn/cn/zh/antibody/product/IgD-Antibody-clone-11-26c-11-26-Monoclonal/63-5993-82).<br>Invitrogen™ eBioscience™ Streptavidin PE Conjugate (https://www.fishersci.no/shop/products/streptavidin-pe-14/11500607).<br>Rabbit anti-HA mAb (https://www.cellsignal.cn/products/primary-antibodies/ha-tag-c29f4-rabbit-mab/3724).<br>Goat anti-rabbit IgG secondary antibody (https://www.thermofisher.cn/cn/zh/antibody/product/Goat-anti-Rabbit-IgG-H-L-Secondary-Antibody-Polyclonal/31460).

Eukaryotic cell lines

Policy information about cell lines and Sex and Gender in Research

Cell line source(s)	Sf9 (Thermo Fisher Scientific # 12659017) and FreeStyle 293F (Thermo Fisher Scientific # R79007) cells were from Thermo Fisher Scientific.
Authentication	None of the cell line used was authenticated.
Mycoplasma contamination	All cell lines were tested negative for mycoplasma contamination.
Commonly misidentified lines<br>(See ICLAC register)	No commonly misidentified cell lines were used.

Palaeontology and Archaeology

Specimen provenance	n/a.
Specimen deposition	n/a.
Dating methods	n/a.

☐ Tick this box to confirm that the raw and calibrated dates are available in the paper or in Supplementary Information.

Ethics oversight	n/a.

Note that full information on the approval of the study protocol must also be provided in the manuscript.

Animals and other research organisms

Policy information about studies involving animals; ARRIVE guidelines recommended for reporting animal research, and Sex and Gender in Research

Laboratory animals	BALB/c female mice (8 week-old; Beijing Vital River Laboratory) were used for immunization. Mice were housed in groups of 3 at specific pathogen free (SPF) status. Photoperiod = 12 hr on: 12 hr off dark/light cycle. Ambient animal room temperature is 23°C, controlled within ± 3°C and room humidity is 50%, controlled within ± 20%.

Wild animals	No wild animals were used in this study.
Reporting on sex	Immune responses can be strongly influenced by biological sex. Klein and colleagues found that female mice show more-robust humoral and cellular immunity than that of male mice after infection with influenza virus. (https://doi.org/10.1073/pnas.1805268115). Therefore, female mice were used for immunization in this study.
Field-collected samples	No field-collected samples were used in this study.
Ethics oversight	All treatments for animals were approved by the Institutional Animal Care and Use Committee (IACUC) of Peking University and conformed to the Guide for the Care and Use of Laboratory Animals (IMM-ChenL-3).

Note that full information on the approval of the study protocol must also be provided in the manuscript.

Clinical data

Policy information about clinical studies

All manuscripts should comply with the ICMJE guidelines for publication of clinical research and a completed CONSORT checklist must be included with all submissions.

Clinical trial registration	n/a.
Study protocol	n/a.
Data collection	n/a.
Outcomes	n/a.

Dual use research of concern

Policy information about dual use research of concern

Hazards

Could the accidental, deliberate or reckless misuse of agents or technologies generated in the work, or the application of information presented in the manuscript, pose a threat to:

No	Yes	
☒	☐	Public health
☒	☐	National security
☒	☐	Crops and/or livestock
☒	☐	Ecosystems
☒	☐	Any other significant area

Experiments of concern

Does the work involve any of these experiments of concern:

No	Yes	
☒	☐	Demonstrate how to render a vaccine ineffective
☒	☐	Confer resistance to therapeutically useful antibiotics or antiviral agents
☒	☐	Enhance the virulence of a pathogen or render a nonpathogen virulent
☒	☐	Increase transmissibility of a pathogen
☒	☐	Alter the host range of a pathogen
☒	☐	Enable evasion of diagnostic/detection modalities
☒	☐	Enable the weaponization of a biological agent or toxin
☒	☐	Any other potentially harmful combination of experiments and agents

Plants

Seed stocks	n/a.
Novel plant genotypes	n/a.
Authentication	n/a.

ChIP-seq

Data deposition

☐ Confirm that both raw and final processed data have been deposited in a public database such as GEO.

☐ Confirm that you have deposited or provided access to graph files (e.g. BED files) for the called peaks.

Data access links *May remain private before publication.*	n/a.
Files in database submission	n/a.
Genome browser session (e.g. UCSC)	n/a.

Methodology

Replicates	n/a.
Sequencing depth	n/a.
Antibodies	n/a.
Peak calling parameters	n/a.
Data quality	n/a.
Software	n/a.

Flow Cytometry

Plots

Confirm that:

☒ The axis labels state the marker and fluorochrome used (e.g. CD4-FITC).

☒ The axis scales are clearly visible. Include numbers along axes only for bottom left plot of group (a 'group' is an analysis of identical markers).

☒ All plots are contour plots with outliers or pseudocolor plots.

☒ A numerical value for number of cells or percentage (with statistics) is provided.

Methodology

Sample preparation	Fresh mouse spleen was obtained, and the isolated spleen was infiltrated in FACS buffer (PBS supplemented with 2% FBS) and transferred to a six-well plate with a 70 µm filter. Grind the spleen to pass through the filter. After depletion of red blood cells (RBCs) by RBC lysis buffer (Thermo Fisher Scientific), splenocytes were resuspend with FACS buffer.
Instrument	BD Aria Fusion
Software	FlowJo_v10.8.1
Cell population abundance	Single cell sort.
Gating strategy	Germinal center B cells were identified as CD4-CD8a-CD19+CD138-B220+CD38-GL7+ population.

☒ Tick this box to confirm that a figure exemplifying the gating strategy is provided in the Supplementary Information.

Magnetic resonance imaging

Experimental design

Design type	n/a.
Design specifications	n/a.
Behavioral performance measures	n/a.

Acquisition

Imaging type(s)	n/a.
Field strength	n/a.
Sequence & imaging parameters	n/a.
Area of acquisition	n/a.

Diffusion MRI ☐ Used ☒ Not used

Preprocessing

Preprocessing software	n/a.
Normalization	n/a.
Normalization template	n/a.
Noise and artifact removal	n/a.
Volume censoring	n/a.

Statistical modeling & inference

Model type and settings	n/a.
Effect(s) tested	n/a.

Specify type of analysis: ☐ Whole brain ☐ ROI-based ☐ Both

Statistic type for inference (See Eklund et al. 2016)	n/a.
Correction	n/a.

Models & analysis

n/a	Involved in the study
☒	☐ Functional and/or effective connectivity
☒	☐ Graph analysis
☒	☐ Multivariate modeling or predictive analysis
